# Visceral fat loss by whole‐body electromyostimulation is attenuated in male and absent in female older Non‐Insulin‐Dependent diabetes patients

**DOI:** 10.1002/edm2.377

**Published:** 2022-10-12

**Authors:** Alexander P. J. Houdijk, Nanneke F. J. M. E. Bos, Wouter M. Verduin, Mick M. Hijdendaal, Michiel A. L. Zwartkruis

**Affiliations:** ^1^ Department of Surgery Northwest Clinics Alkmaar Alkmaar The Netherlands; ^2^ General Practice Bergerhoef Alkmaar The Netherlands; ^3^ General Practice Egmond aan Zee The Netherlands

**Keywords:** electromyostimulation, Type 2 diabetes, visceral obesity

## Abstract

**Introduction:**

Type 2 diabetes and its reversal correlate with increases and decreases in visceral fat (VF). Resistance exercise reduces VF in healthy persons, but little is known in type 2 diabetes. Muscle contractions induced by whole‐body electromyostimulation (WB‐EMS) provide a very effective form of resistance training. We hypothesized that WB‐EMS reduces VF and improves plasma glucose measures in older non‐insulin dependent diabetes mellitus (NIDDM) males and females.

**Methods:**

A four‐arm age‐matched case control study was done on WB‐EMS twice a week in older NIDDM patients (27 males, 18 females) compared with controls (15 males, 15 females). VAT area (VAT, cm^2^), total fat mass (TFM, kg) and lean body mass (LBM, kg) were assessed by DEXA‐scanning. HbA1c, fasting glucose and plasma lipoproteins were measured at baseline and after 4 months.

**Results:**

Baseline control VAT was higher in males than females (140.5 ± 35.6 vs. 96.7 ± 42.3, *p* < .001). In NIDDM, VAT was higher with no significant sex difference (206.5 ± 65.0 vs. 186.5 ± 60.5). In controls, WBEMS reduced VAT in males and females to similar extent (−16.9% and −16.4%, *p* < .001 vs. baseline) and in preference to TFM (−9.2% and −3.6%) or body weight loss (−2.8 and −2.1%). In NIDDM, VF loss was attenuated in males (−7.3%, *p* < .01) but completely absent in females. WBEMS reduced HbA1c and cholesterol and increased HDL levels (all *p* < .05) only in male NIDDM

**Conclusions:**

WBEMS induced VF loss in healthy older males and females an effect strongly attenuated in male and completely absent in female NIDDM patients. This questions the effectiveness of muscle contraction‐induced VF lipolysis in NIDDM. Sex differences may dictate the success of resistance training in NIDDM, a subject that needs to be addressed in future studies.

## Graphical abstract

Our data for the first time show that whole‐body electromyostimulation lowers visceral fat mass in healthy elderly men and women. However, in type 2 diabetes patients this effect was significantly lower in male and completely absent in female patients. Sex differences in muscle‐induced fat lipolysis in type 2 diabetes deserves more attention as this will direct preventive and therapeutic approaches.

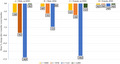

## 1 INTRODUCTION

The global increase in type 2 diabetes has taken pandemic proportions with a predicted prevalence of 570 million patients in 2025.^1^ The lower quality of a reduced lifespan with disturbing increases in healthcare costs urges scientists to search for new, better, and durable preventive and treatment strategies. Accumulating evidence stresses the importance of reducing visceral obesity (VO) in the prevention and treatment of type 2 diabetes as this better reflects disease risk than body weight or BMI.

Visceral obesity initiates a chronic low grade inflammatory state by immune cell influx and a pro‐inflammatory adipokine switch causally linked to atherosclerosis and insulin resistance inducing chronic diseases like hypertension, cardiovascular disease and type 2 diabetes.^2^, ^3^, ^4^ Visceral fat (VF) accumulation is independent of sex, BMI or waist circumference.^5^, ^6^ Reducing VF stimulates efflux of immune cells and anti‐inflammatory adipokine production with restoration of metabolism.^3^, ^7^, ^8^ Sequential VF measurements to monitor success of lifestyle programmes can be done using DEXA with the precision comparable to CT scanning.^9^

Visceral obesity and the risk of developing type 2 diabetes is the consequence of dietary intake of too much of an unbalanced diet and too little physical exercise. Physical exercise programs, even in the absence of calorie restriction reduce VF and lower cardiometabolic and type 2 diabetes risk. A downside is the required training duration and intensity that people find difficult to hold on to. The sedentary untrained type 2 diabetes patient often lacks intrinsic motivation, prematurely leaves programs and is at increased risk for injuries associated with aerobic training.^10^ Strength training therefore is recommended in type 2 diabetes patients as it improves insulin sensitivity and forms a logical starting point to increase muscular strength for future aerobic challenges.^11^ Men have more VF, more glycolytic muscle fibres and are endocrinologically distinct from women who are in the premenopausal, transition or postmenopausal state.^12^ Surprisingly, there is a lack of sex‐controlled reports on physical exercise‐induced VF loss.

A very effective form of strength training is whole‐body electromyostimulation (WB‐EMS) with simultaneous external electrical stimulation of multiple large muscle groups.^13^ WB‐EMS provides muscle contraction without the need of an active movement and is a good alternative for type 2 diabetes patients. Compared with cycling, EMS of even only the legs lengthens the duration of maximal peripheral insulin sensitivity by several hours.^14^ In addition, WB‐EMS augments the higher insulin sensitivity seen after caloric restriction in older obese women with the metabolic syndrome.^15^ Furthermore, 10 weeks of 2 weekly WB‐EMS reduces fasting glucose and HbA1c levels.^16^ However, the effect of WB‐EMS on VF in male and female healthy people and type 2 diabetes patients has not been established.

The purpose of the present study was to investigate the effect of a 4‐months, 2 weekly, WB‐EMS program without calorie restriction, in male and female NIDDM patients (Non‐Insulin‐Dependent Diabetes) on changes in VF, glycaemic control and lipid profile compared with age‐matched controls.

## 2 MATERIALS AND METHODS

### 2.1 Study design, inclusion and exclusion criteria

This prospective four‐arm age‐matched case‐control study on the effect of WB‐EMS was conducted in NIDDM men and women from May 2018 to April 2019. Eligible patients with established NIDDM for more than 2 years and eligible controls willing to participate were recruited by the physical assistants of two general practitioners. Medical history and medication were recorded at baseline. Exclusion criteria were the use of oral antidiabetics other than metformin, prediabetes, epilepsy, hypertension, kidney failure, clinical cardiovascular disease, premenopausal state, oncologic history, chronic obstructive lung disease, anti‐inflammatory medication and a pacemaker. In a study of WB‐EMS, in only 9 diabetes type 2 patients, van Buuren et al. found lower levels of basal glucose and HbA1c.^16^ Because no prior data were available on the effect of WB‐EMS on VF mass, the NIDDM sample size was arbitrarily chosen at least twice this number.

For age matching, the postmenopausal NIDDM women group (*n* = 18) was used for reference because increased age dictates hormonal change that is associated with higher VF mass and an attenuated response to exercise.^17^ Patients recruitment was performed by the general practice nurses who contacted eligible and willing patients within 1 SD around the mean age of 63.3 (±34.1%) of the eligible postmenopausal NIDDM group for possible participation in the study. This resulted in 15 eligible control women, 15 control men and 27 NIDDM men and 18 postmenopausal NIDDM women.

Subjects were not engaged in any other weight loss or physical exercise program and asked to maintain their usual lifestyle during the study. All participants were counselled and encouraged to maintain their regular diet and not change it during the trial. Self‐reported food intake was recorded once weekly. Written informed consent was obtained and the study has been carried out in accordance with the principles of the Declaration of Helsinki as revised in 2008.

### 2.2 Body composition and blood measurements

At baseline, body weight and height were measured, and BMI calculated. Total body mass (TBM, kg), total fat mass (TFM, kg), visceral adipose tissue area (VAT, cm^2^) and lean body mass (LBM, kg) were assessed using dual energy X‐ray absorptiometry (DEXA, Discovery DXA system, Hologic) at baseline and after 4 months.

In the NIDDM group, glucose metabolism was assessed by fasting glucose (mmol/L) and HbA1c (mmol/mol) plasma levels. In addition, plasma lipid profiles were assessed with HDL (mmol/L), LDL (mmol/L) and cholesterol (mmol/L) levels. Blood samples were drawn after a 12 h overnight fast at baseline and after 4 months.

### 2.3 Whole‐body electromyostimulation

EMS provides contraction of agonistic and antagonistic muscles at the same time. Electrodes are attached and energy is transferred transcutaneous to the muscles. The exercise program consisted of 20 min of EMS (MIHA BODYTEC II), twice per week for 4 months. Every session included a 3‐min warming‐up, 15‐min midsection and 2‐min cooling‐down. Four seconds of stimulus were alternated with 4 s of rest. To measure intensity of stimulation, the Borg CR10 scale was used. This scale is rated 0 to 10, corresponding with no exertion noticeable to extremely strong exertion.^18^ In the first session, stimulation corresponding level 4 was delivered, which over time was gradually increased until Borg level 6 was reached. In case of muscle cramps or aches during training, intensity of stimulation could be adjusted. In total, eight muscle groups were stimulated by WB‐EMS: upper arms, chest, upper back, latissimus, abdomen, lower back, buttocks and thighs. All sessions were facility based by certified EMS trainers according to a standardized protocol that limited aerobic exercises to a minimum. Because EMS increases insulin sensitivity, blood glucose levels were taken after each session by finger prick. Patients were instructed to bring a sandwich or banana for consumption after each session.

### 2.4 Statistical analysis

Final analyses included participants with complete data at all timepoints. Data are reported as numbers with percentages for nominal or ordinal variables and means with SDs for continuous variables. Paired sample t‐tests were used to compare baseline and follow‐up data. The significance level was set at *p* < .050. All analyses were performed with SPSS software (v25, IBM Business Analytics; IBM).

## 3 RESULTS

Seventy‐five older participants were enrolled in this study. Forty‐five NIDDM patients were included, 27 males and 18 females and 15 male and 15 female age‐matched controls. During the study, no adverse events or side effects were reported except for two occasions of asymptomatic hypoglycaemia in male NIDDM patients after a WB‐EMS session. The WB‐EMS sessions were well accepted and there were no dropouts.

### 3.1 Demographics and baseline body composition

Table 1 shows the baseline characteristics.

**TABLE 1:** Baseline characteristics for men and women with and without NIDDM.

Baseline characteristics	Male, no NIDDM (*n* = 15)	Male, NIDDM (*n* = 27)	Female, no NIDDM (*n* = 15)	Female, NIDDM (*n* = 18)
Age, years (range)	59.7 (45.0–72.0)	63.0 (45.0–75.0)	59.9 (47.0–65.0)	63.3 (47.0–75.0)
BMI, kg/m^2^ (range)	27.7 (22.8–32.0)	32.3 (22.0–48.2)^a^,**	25.6 (19.0–33.9)	31.1 (21.6–38.9)^b^ ^,#^
Body composition				
TBM, kg	91.3 (12.8)	99.1 (20.2)^a^ ^,^*	71.8 (13.1)^c^ ^,#^	84.1 (17.4)^b^ ^,^*^,^ ^d^ ^,^**
TFM, kg	26.9 (6.2)	31.6 (10.1)	26.8 (7.2)	34.7 (8.5)^b^ ^,^**
VAT, cm^2^	140.5 (35.6)	206.5 (65.0)^a^ ^,^**	96.7 (42.3)^c^ ^,^**	186.5 (60.5)^b^ ^,#^
LBM, kg	64.4 (7.1)	67.5 (10.8)	45.0 (6.2)^c^ ^,#^	49.4 (10.2)^d^ ^,#^
Glucose metabolism				
Glucose, mmol/L	‐	7.8 (2.7)	‐	7.9 (2.7)
HbA1c, mmol/mol	‐	57.1 (12.4)	‐	50.9 (15.2)
%		7.4 (3.3)		6.8 (3.5)
Plasma lipids				
Cholesterol, mmol/L	‐	4.0 (1.1)	‐	4.7 (0.7)
HDL, mmol/L	‐	1.0 (0.3)	‐	1.7 (0.7)
LDL, mmol/L	‐	2.3 (1.0)	‐	2.6 (0.6)

*Note*: Values are presented as means ± SD unless otherwise stated in the table *p < .05, **p < .01, ^#^ *p* < .001.

Abbreviations: HDL, high density lipoprotein; LBM, lean body mass; LDL, low density lipoprotein; TBM, total body mass; TFM, total fat mass; VAT, visceral fat area.

^a^ Men control vs. men NIDDM.

^b^ Women control vs. women NIDDM.

^c^ Men control vs. women control.

^d^ Men NIDDM vs. women NIDDM.

BMI and TBM was higher in NIDDM patients compared with control for both males (*p* < .01 and *p* < .05) and females (*p* < .001 and *p* < .05). All NIDDM patients, except for one female (96 cm^2^), had VAT values higher than 100 cm^2^ above which the risk for cardiovascular disease, diabetes and dyslipidaemia is reported to increase.^19^ In the control group, 13 males and six females were VO with the usual higher VAT in males (*p* < .01). Compared with their sex controls, VAT was significantly higher in the NIDDM group (47% in male *p* < .01 and 93% in female *p* < .001) with no sex difference. In females, TFM was significantly higher in NIDDM compared with control (*p* < .01). LBM did not differ between controls and NIDDM. The common higher LBM in males was found in both the NIDDM (*p* < .001) and control group (*p* < .001).

### 3.2 WB‐EMS effect on body composition

Table 2 shows the intra group comparisons for changes in body composition after 4 months of WBEMS. Total body mass decreased in all groups (*p* < .05) except for NIDDM females. Reductions in TFM were seen in male and female controls (*p* < .01 and *p* < .05) and male NIDDM (*p* < .05) with no effect in NIDDM females. Visceral fat area reduced in control males (*p* < .001) and females (*p* < .0001) and NIDDM males (*p* < .0001) with no change in female NIDDM patients. In all groups, LBM did not change during the 4 months training period.

**TABLE 2:** Body composition after WB‐EMS versus baseline for control and NIDDM men and women.

	Male, control		Male, NIDDM		Female, control		Female, NIDDM	
	Baseline	+4 months	Baseline	+4 months	Baseline	+4 months	Baseline	+4 months
TBM, kg	91.3 (12.8)	88.5 (12.1)*	99.1 (20.2)	98.0 (20.0)*	71.8 (13.1)	70.1 (12.2)*	84.1 (17.4)	83.9 (18.5)
TFM, kg	26.9 (6.2)	24.3 (6.0)**	31.6 (10.1)	30.6 (10.3)*	26.8 (7.2)	25.8 (7.0)*	34.7 (8.5)	34.6 (8.7)
VAT, cm^2^	140.5 (35.6)	116.6 (37.0)***	206.5 (65.0)	189.9 (56.6)^#^	96.7 (42.3)	84.1 (42.8)^#^	186.5 (60.5)	185.5 (67.9)
LBM, kg	64.4 (7.1)	64.2 (6.66)	67.5 (10.8)	67.4 (10.3)	45.0 (6.2)	44.3 (5.3)	49.4 (10.2)	49.3 (10.8)

*Note*: Change after 4 months WBEMS versus baseline. Values are presented as means ± SD.

Abbreviations: LBM, lean body mass; TBM, total body mass; TFM, total fat mass; VAT, visceral fat area.

* *p* < .05.

** *p* < .01.

*** *p* < .001.

^#^ *p* < .0001.

**FIGURE 1:**
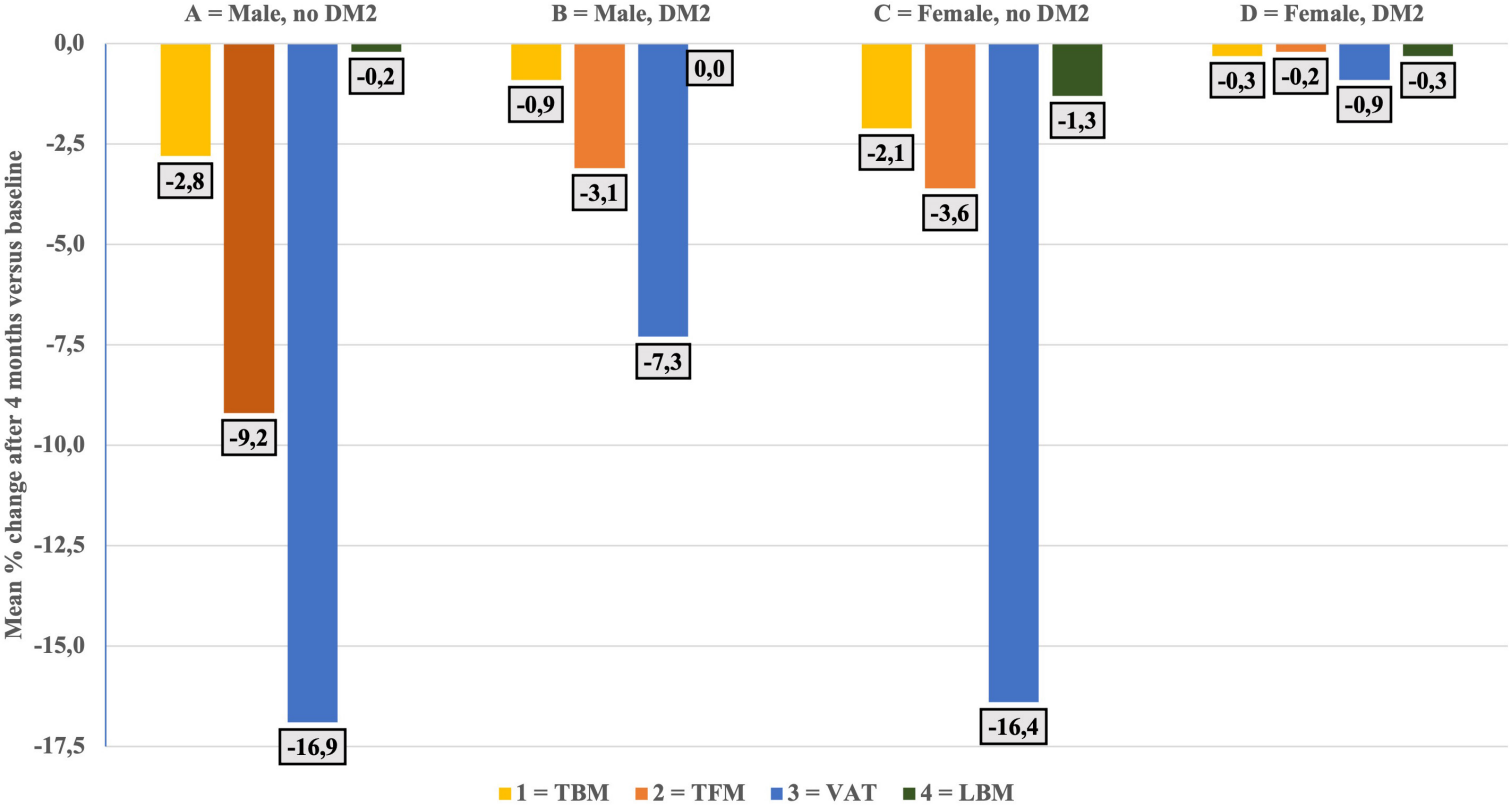
Percentual decreases are shown for intragroup and intergroup changes. *Male control*; VAT vs. TFM *p* < .01, VAT vs. TBM *p* < .0001, TFM vs. TBM *p* < .001. *Male NIDDM*; VAT vs. TFM *p* < .001, VAT vs. TBM *p* < .0001, TFM vs. TBM *p* < .01. *Female control*; VAT vs. TFM *p* < .01, VAT vs. TBM *p* < .01. *Male control vs. male NIDDM*; VAT *p* < .001, TFM *p* = .01. *Male NIDDM vs. female NIDDM*; TBM *p* < .05, VAT *p* < .05. *Female control vs. Female NIDDM*; VAT *p* < .005.

Figure 1 shows the percentual changes in body composition for intra‐ and intergroup comparison. Similar percentage reductions in VAT were seen in male and female controls (−16.9% and −16.4%) with lower reductions for TFM (males −9.2% *p* < .01; females −3.6%, *p* < .01 vs. VAT) and for TBM (males −2.8% *p* < .0001; females −2.1% *p* < .01 vs. VAT). In the NIDDM group, males had a lower loss of VAT compared with male controls (−7.3% *p* < .01) but greater relative to that of TFM (−3.1% *p* < .001) and TBM (−0.9% *p* < .0001). The loss of TBM and VFA in male NIDDM was significantly different from that in NIDDM females (both *p* < .05). The VAT loss in control females was higher compared with NIDDM females (*p* < .005).

### 3.3 WB‐EMS effect on plasma glucose and lipid parameters in NIDDM

Table 3 shows the effect of 4 months of WB‐EMS on plasma parameters of glucose metabolism and lipids in male and female NIDDM. Changes were only noted in male NIDDM patients. Fasting glucose did not change, but HbA1c decreased from 55.7 ± 12.7 mmol/mol to 52.7 ± 13.8 mmol/mol (*p* < .05). Cholesterol reduced from 4.0 ± 0.7 to 3.6 ± 0.6 mmol/L (*p* < .05) and HDL increased from 1.0 ± 0.3 mmol/L to 1.2 ± 0.3 mmol/L (*p* = .05). LDL showed no change (*p* = .247).

**TABLE 3:** Plasma derivatives for glucose and lipid metabolism after WB‐EMS in NIDDM men and women.

	Male, no NIDDM		Male, NIDDM		Female, no NIDDM		Female, NIDDM	
	Baseline	+4 months	Baseline	+4 months	Baseline	+4 months	Baseline	+4 months
Glucose, mmol/L	‐	‐	8.9 (2.6)	8.5(3.3)	‐	‐	8.3 (2.8)	8.1 (2.9)
HbA1c, mmol/mol	‐	‐	55.7 (12.7)	52.7 (13.8)*	‐	‐	51.1 (15.7)	51.3 (12.5)
Cholesterol, mmol/L	‐	‐	4.0 (0.7)	3.6 (0.6)*	‐	‐	4.7 (0.7)	4.7 (0.7)
HDL, mmol/L	‐	‐	1.0 (0.3)	1.2 (0.3)*	‐	‐	1.7 (0.7)	1.5 (0.4)
LDL, mmol/L	‐	‐	2.3 (0.7)	2.2 (0.6)	‐	‐	2.6 (0.7)	2.6 (0.7)

*Note*: Change after 4 months WBEMS versus baseline. Values are presented as means ± SD. Abbreviations: HDL, high density lipoprotein; LDL, low density lipoprotein.

* *p* < .05.

## 4 DISCUSSION

For the first time, we show that WB‐EMS can reduce VF in healthy older men and women. Visceral fat was preferentially reduced compared with TFM and TBM. In NIDDM, the reduction of VF was strongly attenuated in male and completely absent in female patients. In NIDDM, VF was much higher than in controls with the loss of the common sex difference of higher VF in men as seen in controls. Plasma derivatives of glucose and lipid metabolism were improved in male but not in female NIDDM patients.

The accumulation of VF is a key factor in the pathogenesis of overweight‐associated chronic diseases like hypertension, cardiovascular disease and type 2 diabetes.^2^, ^20^ It induces an atherogenic lipoprotein profile and sets of a chronic inflammatory state by influx and retention of macrophages and lymphocytes in VF that is causally related to insulin resistance and atherosclerosis.^3^, ^4^ Reducing VF ameliorates dyslipidaemia and stimulates efflux of immune cells thereby improving insulin sensitivity and metabolic risk factors.^7^

Throughout life, men have more VF than women as seen in our control group.^12^ The NIDDM patients had significantly higher VAT's, but the VF sex difference had disappeared. Indeed, compared with control the NIDDM women had accumulated more VF than men (93% vs. 47%). This implies that the NIDDM women had stored VF more rapidly catching up with men. Our data agree with Goodpaster et al. who reported on worsening of glucose tolerance with increasing VFA's. They showed that in normal and impaired glucose tolerance, men had higher VAT's than women (145 ± 66 vs. 116 ± 54 and 163 ± 72 vs. 141 ± 60 cm^2^ resp. *p* < .0001) but here was no difference for the highest VFA's in NIDDM patients (172 ± 79 vs. 162 ± 66 cm^2^).^21^ With the loss of oestrogen and reduced energy expenditure, postmenopausal women accumulate VF at a faster rate with inflammatory changes and increased cardiometabolic disease risk.^17^ However, the postmenopausal state per se cannot explain the absence of the VF sex difference in NIDDM because in the postmenopausal control women VF was lower than in men. The (pre) NIDDM state probably leads to the rise in VF accumulation catching up with men. The rapid increase in VF in postmenopausal women may underlie some of the reported greater disease risks compared with men. In a study on the causal role of VF on disease risk in older men and women, a striking higher risk for NIDDM was found in women (OR 7.34 vs. 2.5).^22^

Systematic reviews and meta‐analyses on VF loss by physical exercise show that in the healthy obese or non‐obese intensive exercise is effective with a preference for high intensity aerobic or combined training modalities.^23^ The untrained type 2 diabetes patient is at greater risk for cardiac events and injury by aerobic exercise and initially resistance training is recommended.^11^ As a powerful resistance training, WB‐EMS is a safe and time‐efficient alternative. The external muscle contractions are evoked in a standardized manner enabling better individual and group comparison because individual differences that come with executing active forms of exercise are avoided. This is especially relevant when comparing a sedentary NIDDM group with a healthy control group that most likely has a more active lifestyle. During the WB‐EMS sessions, aerobic exercises therefore were kept to a minimum.

For the first time, we show that 16 weeks of WB‐EMS lowered VF by almost 17% both in healthy older men and postmenopausal women. This is quite remarkable realizing that men and women differ in expression of more than 3000 muscle genes, men have higher muscle mass, more glycolytic fast twitching type IIa fibres and muscle and fat physiology is regulated differently.^24^ To our knowledge, there is only one sex comparative study by Janssen et al. who also reported no sex differences of VF loss between healthy obese men and premenopausal women in response to diet, or diet combined with either aerobic or resistance exercise.^25^ In contrast to our findings, however, Kemmler et al. after 12 months WB‐EMS found no change in abdominal fat measured with DEXA in sedentary osteopenic older women with central obesity. Abdominal fat however was not further specified into VF or subcutaneous fat. The latter is reduced to a lesser extent as confirmed by our TFM data and differences in VF may have been missed. In addition, the older age (75 ± 4 year), less frequent sessions (3/2 weeks) and control for food intake only at the beginning and end may also underlie the lack of any effect in their study.^26^ Overall, our WB‐EMS effect on VF loss in healthy controls is in line with exercise literature.

WB‐EMS induced a preferential loss of VF compared with TFM in both control men and women (Figure 1) a finding that confirms results of various types of exercise and (very) low calorie diets.^27^ This may be explained by the reported greater muscle‐induced β1 and β2 catecholamine‐induced lipolysis and lower antilipolytic insulin action in visceral adipocytes from non‐obese subjects compared with subcutaneous fat.^28^ In our male and female controls, adrenergic responses on WB‐EMS probably are similar but this needs to be verified.

The WB‐EMS‐induced VF loss was significantly attenuated in NIDDM men and remarkably no effect at all was seen in women. The ability of physical exercise to reduce VF in type 2 diabetes has been reviewed by Sabag et al. who concluded that especially aerobic exercise reduces VF but also stated that the lack of controlled cohort studies precludes conclusions on attenuation of VF loss or sex differences in type 2 diabetes.^29^ To our knowledge, the only study on exercise‐induced VF loss in NIDDM using a sex control group is by Dobrosielsky et al. They retrospectively combined data from 2 different trials of patients with hypertension with or without NIDDM and reported a strongly attenuated VF loss in the NIDDM males (−23% vs. −2%) and females (−13% vs. −4%), with no sex difference. Combining data from two different trials retrospectively, no age‐matched controls, presence of hypertension, differences in their NIDDM male to female VAT's (177 vs. 120 cm^2^) and unexplained higher VAT in male controls (187 cm^2^) may have obscured finding a sex difference.^30^

Overall, our data and that of others indicate an attenuation of exercise‐induced lipolysis of VF in NIDDM that is not explained in literature. On the possible underlying mechanisms, especially for the complete absence of an effect in the NIDDM women, we can only speculate. However, the adrenergic pathway and the myokine Interleukin 6 (IL‐6)‐induced muscle‐fat crosstalk deserve mentioning. Obese men have higher VF lipolysis by upregulation of β3 adrenoreceptors and lower antilipolytic α2 adrenergic function.^28^ This may be involved in the sex difference in VF loss in the obese NIDDM group. The myokine IL‐6 is strongly linked to VF lipolysis because injecting Tociluzimab, an IL‐6 receptor blocking antibody completely blocks VF lipolysis after ergometric cycling.^31^ The attenuation (men) and absence of VF loss (women) after WB‐EMS in NIDDM may relate to lower muscle production or VF response to IL‐6. Another possibility is that in NIDDM greater muscle stimuli are needed in male and even greater in females to induce VF lipolysis to reach control levels. The combination of simultaneous WB‐EMS and aerobic exercise and/or calorie restriction may be an attractive alternative for reducing VF in the NIDDM men and women that needs to be explored.

Our WB‐EMS protocol had no effect on LBM, a finding not supported by the increase reported by Kemmler et al.^13^ In their meta‐analysis, a significant increase was noted in muscle mass measured with bioimpedance, leg circumference and DEXA. We have no clear explanation for this discrepancy but the absence of exercises in our WB‐EMS protocol may have limited muscle growth.

The loss of VF in the NIDDM men coincided with lowering of HbA1c, an increase in HDL and lowering of cholesterol levels suggesting metabolic improvement. In NIDDM women, there were no changes in the metabolic parameters. Reljic et al. already showed that WB‐EMS lowers cholesterol in obese elderly women with the metabolic syndrome,^32^ and significant reductions of HbA1c were seen after 10 weeks of WB‐EMS.^14^ We did not find the lower fasting blood glucose levels that van Buuren et al. reported after WB‐EMS in a small mixed sex type 2 diabetes population.^16^ This is probably explained by the higher fasting basal glucose levels of 9.1 mMol/L in their more heterogenic type 2 diabetes group on glicazide and insulin medication.

Strengths and limitations of our study need to be addressed. A strong quality was the four‐arm prospective case control design exposing the main study effects of the sex differences in NIDDM compared with controls. Other strengths are related to the use of WB‐EMS as a highly standardized and reproducible form of resistance exercise minimizing influences of individual motivation maximizing group comparison. Furthermore, only metformin was allowed as an oral antidiabetic contributing to the quality of comparison. A limitation is the use of self‐reported dietary records as they may underestimate real food intake, especially in the female NIDDM group. However, there was no increase in VF in NIDDM females during the study something expected with increased caloric intake. Another limitation is that muscle strength as an outcome parameter for WB‐EMS was not measured. Strength building is considered more important in metabolic change than LBM that was not different between the groups. Differences in strength might have provided more insight in the different WB‐EMS effects. The method of age‐matching groups with the use of 1 SD around the mean of the NIDDM postmenopausal women allowed for recruiting patients and controls from a distinct geographical setting of two general practices. It might have been better to have a one‐on‐one age match, but this was not practical. Our results are of an observational nature and should be interpreted as such. Our assumption that no abnormalities in plasma derivatives of glucose and lipid metabolism were present in the healthy controls and for that reason not measured might have been false and relevant changes in response to WB‐EMS been missed.

In conclusion, our results show the effectiveness of WBEMS on reducing VF in healthy older men and women and provide an efficient option for primary prevention of VF‐related diseases. The attenuation of the WB‐EMS effect in type 2 diabetes men and complete absence in women is a troublesome unprecedented finding. Our data stress the importance of sex‐specific approaches for reducing VF loss in NIDDM. The void of sex‐controlled studies on the mechanisms of exercise‐induced VF loss in type 2 diabetes needs to be filled quickly to better direct preventive and therapeutic strategies.

## AUTHOR CONTRIBUTIONS

**Nanneke Bos:** Data curation (lead); formal analysis (lead); investigation (lead); writing – original draft (lead). **Wouter Verduin:** Data curation (lead); formal analysis (lead); investigation (lead); methodology (equal); validation (equal); visualization (equal). **Mick Hijdendaal:** Data curation (lead); supervision (equal); validation (equal); writing – review and editing (equal). **Michiel Zwartkruis:** Conceptualization (lead); data curation (equal); investigation (equal); supervision (lead); validation (equal); writing – review and editing (equal).

## FUNDING INFORMATION

This research received no specific grant from any funding agency in the public, commercial or not‐for‐profit sectors.

## CONFLICT OF INTEREST

N.B., W.V., M.H. and M.Z. have no relevant conflict of interest. A.H. is co‐founder and CEO of Preventimed, a research start‐up company originating from a grant from the European Regional Development Fund focused on prevention and treating visceral obesity associated diseases.

## Data Availability

Data are available on request from the authors.
